# SIRT1 in Cardiac Diseases: Molecular Mechanisms, Therapeutic Potential, and Future Directions

**DOI:** 10.3390/ijms27104216

**Published:** 2026-05-09

**Authors:** Yingxuan Chang, Le Li, Hongmei Yue

**Affiliations:** Department of Respiratory Medicine, The First Hospital of Lanzhou University, Lanzhou 730000, China; changyx0019@163.com (Y.C.); c9422880515@gmail.com (L.L.)

**Keywords:** SIRT1, heart failure, myocardial ischemia–reperfusion injury, diabetic cardiomyopathy, cardiac hypertrophy, ferroptosis, mitochondrial biogenesis, natural compounds

## Abstract

Sirtuin 1 (SIRT1), a nicotinamide adenine dinucleotide (NAD^+^)-dependent class III histone deacetylase, functions as a central metabolic sensor and stress-responsive regulator in the cardiovascular system. Unlike its well-characterized role in atherosclerosis, SIRT1 exerts multifaceted protective effects directly on cardiac tissue. This review synthesizes recent advances in understanding SIRT1-mediated cardioprotection across a spectrum of heart diseases, including myocardial ischemia/reperfusion (I/R) injury, heart failure (HF), diabetic cardiomyopathy (DCM), cardiac hypertrophy, aging-related cardiac dysfunction and circadian rhythm disruption. Mechanistically, SIRT1 orchestrates antioxidant defense through nuclear factor erythroid 2-related factor 2 (Nrf2) and Forkhead box O (FoxO) transcription factors activation, suppresses inflammatory signaling via nuclear factor kappa B (NF-κB) deacetylation, inhibits apoptosis by targeting p53, promotes autophagic flux and mitophagy, regulates mitochondrial biogenesis through peroxisome proliferator-activated receptor gamma coactivator 1-alpha (PGC-1α), and controls ferroptosis via the Nrf2/glutathione peroxidase 4 (GPX4) axis. Preclinical studies demonstrate that natural compounds (resveratrol, quercetin, curcumin, ginsenosides, tanshinone IIA, bergenin, swietenine) and synthetic SIRT1 activators (SRT1720, anilinopyridine derivatives) attenuate cardiac injury and improve function. Moreover, SIRT1 serves as a prognostic biomarker in HF and diabetic patients. However, context-dependent dual roles, where excessive SIRT1 expression may be detrimental, underscore the need for precise modulation. Challenges remain in achieving cardiac-specific targeting, optimizing NAD^+^ availability, and translating preclinical findings into clinical practice. Future research should integrate multi-omics approaches, single-cell transcriptomics, and precision medicine strategies to unlock the therapeutic potential of SIRT1 in cardiac diseases.

## 1. Introduction

Cardiovascular diseases (CVDs) remain the leading cause of global morbidity and mortality, with heart failure (HF), myocardial infarction, and diabetic cardiomyopathy (DCM) accounting for a substantial proportion of this burden [1,2,3]. While atherosclerosis contributes significantly to coronary artery disease, the heart itself undergoes complex pathological remodeling driven by oxidative stress, inflammation, metabolic dysregulation, and cell death programs that are not fully captured by vascular-focused models [4,5]. Over the past two decades, Sirtuin 1 (SIRT1), a nicotinamide adenine dinucleotide (NAD^+^)-dependent deacetylase, has emerged as a critical integrator of cellular stress responses in the myocardium [5,6].

SIRT1 belongs to the sirtuin family (SIRT1–SIRT7), which is characterized by its dependence on NAD^+^ as a cofactor [7,8]. By deacetylating both histone and non-histone proteins, SIRT1 regulates diverse biological processes, including energy metabolism, oxidative stress resistance, inflammation, autophagy, apoptosis, and mitochondrial function [9,10,11,12]. In the heart, SIRT1 expression and activity are modulated by nutritional status, exercise, and various pathological stimuli [13,14]. Importantly, SIRT1 exhibits a dose-dependent dual role: low-to-moderate overexpression confers cardioprotection, whereas high levels can induce cardiomyopathy [6,13,15].

Rather than functioning as a simple on/off switch, SIRT1 is better conceptualized as a metabolic rheostat that continuously tunes cellular responses. Its activity is dynamically regulated by NAD^+^ availability, the intensity and duration of cellular stress, and the stage of disease progression [6,13]. This rheostat-like behavior provides a unifying framework to reconcile the dose- and context-dependent effects of SIRT1 observed across different cardiac conditions.

Beyond its well-established role in pathological conditions, SIRT1 also plays a critical role in cardiac development and maturation. Emerging evidence indicates that SIRT1 regulates cardiomyocyte differentiation, structural organization, and metabolic remodeling during heart development. SIRT1 knockout in human embryonic stem cells significantly downregulates mesodermal, cardiac precursor, and mature cardiomyocyte markers, demonstrating its essential role in cardiomyogenesis [16]. Through deacetylation of key transcription factors such as peroxisome proliferator-activated receptor gamma coactivator 1-alpha (PGC-1α) and Forkhead box O (FoxO) transcription factors, SIRT1 promotes mitochondrial maturation and the transition toward oxidative metabolism, which is essential for postnatal cardiac function [17]. In addition, SIRT1 activity is tightly controlled through interactions with other sirtuin family members and epigenetic regulators, highlighting its role in coordinating differentiation programs at multiple levels. Recent studies further demonstrate that SIRT1 is indispensable for cardiomyocyte alignment and structural maturation. SIRT1 deficiency disrupts myofibrillar organization and impairs chemotactic signaling pathways, including CXCL12/CXCR4 and CCL2/CCR2, leading to abnormal cardiac architecture and reduced heart size [18]. Proteomic profiling further reveals that cardiomyocyte maturation relies on Sirtuin signaling pathway-mediated metabolic remodeling [19]. Collectively, these findings establish SIRT1 not only as a metabolic and stress-response regulator but also as an orchestrator of cardiomyocyte remodeling and functional maturation during cardiac development. Thus, SIRT1 functions as a key regulator spanning cardiac development, maturation, and disease progression, reinforcing its potential as a therapeutic target across the lifespan.

While the role of SIRT1 in vascular biology and atherosclerosis has been extensively reviewed elsewhere [20], this review focuses specifically on direct cardiac actions of SIRT1. We synthesize evidence from preclinical models and clinical studies, discuss molecular mechanisms across different cardiac pathologies, evaluate therapeutic strategies targeting SIRT1, and highlight unresolved questions and future directions.

## 2. Molecular Mechanisms of SIRT1 in Cardiac Protection

**Figure 1:**
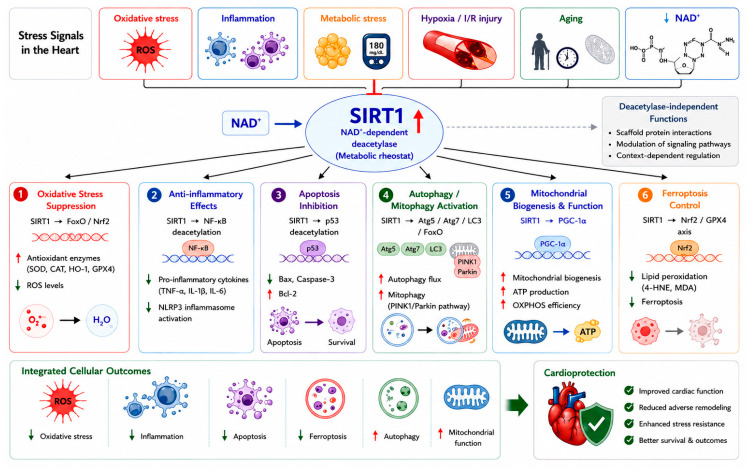
Molecular mechanisms of SIRT1 in cardiac protection. SIRT1, a NAD^+^-dependent deacetylase, integrates multiple cardiac stress signals to regulate key protective pathways. It enhances antioxidant defense, suppresses inflammation and apoptosis, promotes autophagy and mitochondrial function, and inhibits ferroptosis through targets such as FoxO, NF-κB, p53, and PGC-1α. These coordinated actions reduce cellular damage and improve cardiac function, contributing to overall cardioprotection. Red arrows mean increase/activation/upregulation; Green arrows mean decrease/inhibition/downregulation.

SIRT1 protects the heart through an integrated network of downstream effectors. These mechanisms are not mutually exclusive but rather operate in a coordinated, cell-type- and context-dependent manner (Figure 1).

### 2.1. Oxidative Stress Suppression

Oxidative stress is a common driver of cardiac injury. SIRT1 enhances antioxidant defenses by deacetylating and activating Forkhead box O (FoxO) transcription factors, which upregulate manganese superoxide dismutase (MnSOD) and catalase [21,22]. In a mouse model of myocardial ischemia/reperfusion (I/R) injury, cardiac-specific SIRT1 overexpression reduced oxidative stress markers and infarct size through FoxO-dependent mechanisms [22]. SIRT1 also activates nuclear factor erythroid 2-related factor 2 (Nrf2), a master antioxidant regulator. Studies have shown that SIRT1 directly deacetylates Nrf2, promoting its nuclear translocation and transcriptional activity, leading to increased expression of heme oxygenase-1 (HO-1), NAD(P)H quinone oxidoreductase 1 (NQO1), and glutathione peroxidase 4 (GPX4) [23,24,25]. In DCM, resveratrol enhances SIRT1-dependent antioxidant signaling, leading to reduced ROS levels and improved cardiac function [25].

### 2.2. Anti-Inflammatory Effects

Chronic inflammation contributes to cardiac remodeling and HF progression. SIRT1 inhibits the pro-inflammatory nuclear factor kappa B (NF-κB) pathway by deacetylating the RelA/p65 subunit at lysine 310, thereby reducing transcription of TNF-α, IL-1β, IL-6, and other cytokines [26,27]. In a rat model of PM2.5-induced cardiovascular inflammation, phytosome-encapsulated Zea mays extract upregulated SIRT1 and suppressed NF-κB activation, ameliorating cardiac inflammation [28]. In DCM, swietenine, a limonoid compound, suppressed NLRP3 inflammasome activation via the NAMPT/SIRT1 pathway, reducing myocardial hypertrophy and fibrosis [29]. Similarly, tanshinone IIA alleviated endoplasmic reticulum stress-mediated inflammation in diabetic hearts through SIRT1 upregulation [30].

### 2.3. Apoptosis Inhibition

Cardiomyocyte apoptosis is a key determinant of cardiac dysfunction. SIRT1 deacetylates and inactivates p53, thereby reducing Bax expression and caspase-3 activation while preserving anti-apoptotic Bcl-2 [31,32]. In a myocardial infarction (MI) model, mangiferin prevented cardiomyocyte apoptosis and subsequent HF by activating the SIRT1/FoxO3a pathway [31]. Curcumin attenuated oxidative stress and apoptosis in DCM through SIRT1-FoxO1 and PI3K-Akt signaling [32]. Indobufen, an antiplatelet drug, reduced myocardial I/R injury via the PI3K/Akt/eNOS pathway, with SIRT1 playing a permissive role [33]. The natural formulation IQ-RKT (containing rutin, kaempferol, and thymoquinone) inhibited AGEs-induced cardiomyocyte apoptosis by targeting the AGE-RAGE-ROS-dependent TRAF3IP2/JNK axis, with SIRT1 involvement suggested [34]. These findings indicate that SIRT1-mediated regulation of apoptosis represents one component of a broader network of cell death pathways, which also includes autophagy- and ferroptosis-related mechanisms, as discussed below.

### 2.4. Autophagy and Mitophagy Regulation

Autophagy is essential for removing damaged organelles and maintaining cardiomyocyte homeostasis. SIRT1 promotes autophagy by deacetylating key autophagy-related proteins (Atg5, Atg7, LC3) and FoxO transcription factors [35,36,37]. In HF following MI, ginsenoside Rg1 protected against cardiac remodeling by enhancing SIRT1/PINK1/Parkin-mediated mitophagy [36]. Dexmedetomidine, an anesthetic, alleviated myocardial I/R injury by restoring SIRT1 expression and activating FoxO3a, thereby modulating autophagy and apoptosis [35]. MLN4924, a neddylation inhibitor, exerted cardioprotective effects by restoring autophagic flux in a SIRT1-dependent manner [37]. Quercetin improved cardiomyocyte vulnerability to hypoxia by regulating SIRT1/TMBIM6-related mitophagy and endoplasmic reticulum stress [38]. Together with apoptosis and ferroptosis, SIRT1-dependent regulation of autophagy contributes to an integrated network controlling cardiomyocyte survival and stress adaptation. In addition to apoptosis, autophagy, and ferroptosis, emerging forms of regulated cell death such as cuproptosis have recently been implicated in cardiovascular pathology, suggesting an expanded and evolving landscape of SIRT-related cell death regulation [39].

### 2.5. Mitochondrial Biogenesis and Function

Mitochondrial dysfunction is central to cardiac pathology. SIRT1 deacetylates and activates PGC-1α, a master regulator of mitochondrial biogenesis, leading to increased mitochondrial content, enhanced oxidative phosphorylation, and improved ATP production [14,40,41,42]. Nicotinamide riboside (NR), a NAD^+^ precursor, promoted Mfn2-mediated mitochondrial fusion in diabetic hearts through the SIRT1-PGC1α-PPARα pathway, reducing cardiomyocyte apoptosis [41]. Bergenin alleviated myocardial I/R injury by activating the SIRT1/AMP-activated protein kinase (AMPK)/PGC-1α pathway and inhibiting ferroptosis [43]. CTRP6 (C1q/TNF-related protein 6) ameliorated HF via the AMPK/SIRT1/PGC-1α signaling pathway [40]. Resveratrol-induced SIRT1 activation by LKB1-mediated phosphorylation enhanced mitochondrial biogenesis and respiration through PGC-1α deacetylation [14].

### 2.6. Ferroptosis Control

In addition to apoptosis and autophagy, ferroptosis has recently emerged as a distinct form of regulated cell death in cardiovascular diseases. Ferroptosis, an iron-dependent form of regulated cell death distinct from apoptosis, has recently been implicated in I/R injury, HF, and DCM. SIRT1 suppresses ferroptosis through multiple mechanisms. Resveratrol inhibited ferroptosis and decelerated HF progression via the SIRT1/p53 pathway by reducing SLC7A11 degradation and maintaining GPX4 levels [25]. Bergenin exerted protective effects against I/R injury by modulating ferroptosis through the SIRT1/AMPK/PGC-1α pathway [43]. Paclitaxel, though primarily studied in atherosclerosis, attenuated macrophage ferroptosis via the SIRT1/Nrf2/GPX4 pathway, a mechanism that may extend to cardiac macrophages [23]. Ilexgenin A alleviated myocardial I/R injury by inhibiting ferroptosis through the SIRT1 pathway [44]. ALKBH5-mediated m^6^A demethylation protected against myocardial I/R injury via FSP1-dependent inhibition of ferroptosis, with SIRT1 playing a regulatory role [45]. Collectively, SIRT1 regulates multiple forms of programmed cell death, including apoptosis, autophagy, and ferroptosis, highlighting its role as a central coordinator of cell fate decisions in cardiac pathology.

### 2.7. Deacetylase-Independent Functions

In addition to its well-characterized NAD^+^-dependent deacetylase activity, SIRT1 also exerts important deacetylase-independent functions that contribute to its regulatory versatility. Emerging evidence suggests that SIRT1 can act as a scaffold protein, facilitating protein–protein interactions and modulating transcriptional complexes independently of its catalytic activity. For example, SIRT1 has been shown to interact with key transcription factors and co-regulators to influence gene expression without directly deacetylating substrates [10,31]. Moreover, SIRT1 may regulate signaling pathways through structural or allosteric mechanisms, thereby influencing cellular processes such as stress responses, mitochondrial homeostasis, and metabolic adaptation. In certain contexts, catalytically inactive SIRT1 mutants retain partial biological activity, further supporting the existence of non-enzymatic functions [38]. These findings indicate that SIRT1 functions not only as an enzymatic regulator but also as a multifunctional signaling hub. Importantly, the balance between catalytic-dependent and -independent mechanisms may determine the overall biological outcome of SIRT1 activation. This dual functionality may partly explain the context-dependent effects of SIRT1 observed in cardiovascular diseases and highlights the need for more precise therapeutic strategies targeting both enzymatic and non-enzymatic functions.

## 3. SIRT1 in Specific Cardiac Diseases

**Figure 2:**
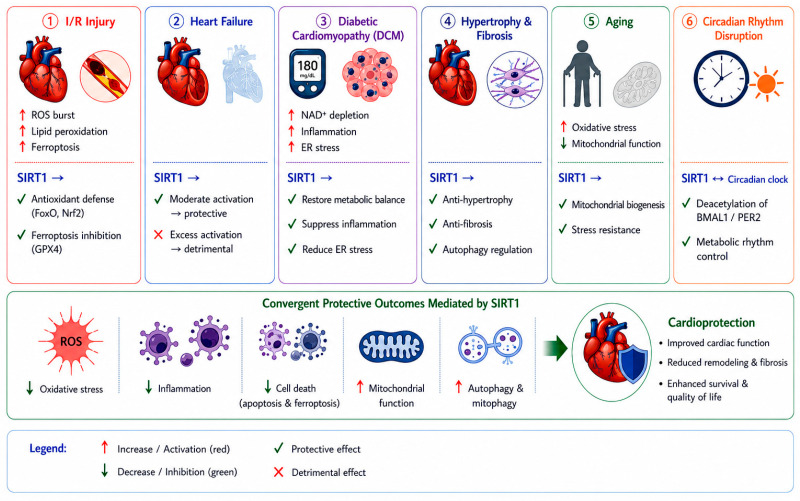
Disease-specific roles of SIRT1 in cardiac pathophysiology. SIRT1 regulates multiple cardiac diseases in a context-dependent manner. It reduces oxidative stress, inflammation, and cell death while enhancing mitochondrial function and autophagy. Its effects vary across conditions such as I/R injury, heart failure, DCM, hypertrophy, aging, and circadian disruption, but collectively converge to improve cardiac function and promote cardioprotection.

Building on these molecular mechanisms, the cardioprotective roles of SIRT1 become more apparent in specific pathological contexts. In the following sections, we summarize how SIRT1 functions across major cardiac diseases, highlighting its context-dependent effects and therapeutic relevance (Figure 2).

### 3.1. Myocardial I/R Injury

I/R injury is characterized by a rapid burst of ROS and lipid peroxidation upon reperfusion, leading to acute cardiomyocyte damage. In this context, SIRT1 primarily exerts cardioprotective effects by attenuating oxidative stress and regulating ferroptosis [13,22]. SIRT1-dependent antioxidant pathways enhance cellular defense and limit ROS accumulation, thereby reducing lipid peroxidation and preserving membrane integrity [46]. Recent studies further indicate that ferroptosis represents a dominant mode of cell death in I/R injury [45]. SIRT1 modulates ferroptotic pathways through regulation of downstream targets such as GPX4 and lipid metabolism-related processes, thereby suppressing iron-dependent oxidative injury [25,43,44]. Unlike chronic cardiac conditions, where metabolic remodeling is predominant, the protective role of SIRT1 in I/R injury is largely centered on acute redox balance and ferroptosis control. Ginsenoside Rb2 has been reported to alleviate myocardial I/R injury, partly through engagement of the SIRT1 signaling axis [47]. However, its cardioprotective effects are not limited to SIRT1 signaling, as Rb2 also modulates multiple pathways involved in inflammation and oxidative stress, including NF-κB and Nrf2 signaling [48]. These findings suggest that the beneficial effects of Rb2 arise from coordinated regulation of multiple molecular targets rather than a single pathway. These observations highlight the complexity of natural compounds, which often exert pleiotropic effects through interconnected signaling networks, thereby complicating the attribution of their therapeutic actions to a single molecular target.

### 3.2. HF

HF is characterized by progressive structural remodeling and metabolic dysregulation. In contrast to I/R injury, the role of SIRT1 in HF is highly context-dependent and influenced by expression levels and duration of activation. Moderate engagement of SIRT1-dependent metabolic signaling (2.5–7.5-fold) has been shown to preserve mitochondrial function, enhance energy metabolism, and attenuate pathological hypertrophy. However, excessive SIRT1 axis engagement (~12.5-fold) may induce mitochondrial dysfunction and contribute to cardiomyopathy, highlighting a dose-dependent duality [13]. These observations are consistent with the concept of SIRT1 as a metabolic rheostat, where both insufficient and excessive activity can lead to adverse outcomes. Mechanistically, SIRT1 in HF primarily regulates metabolic remodeling, including mitochondrial biogenesis and substrate utilization, rather than acute oxidative stress responses [25,40,49]. Thus, SIRT1 acts as a metabolic regulator in HF, and its therapeutic targeting requires precise modulation to avoid maladaptive effects.

### 3.3. DCM

DCM is driven by chronic metabolic stress, insulin resistance, and persistent inflammation. A central feature of DCM is NAD^+^ depletion, which directly impairs SIRT1 activity and disrupts cellular homeostasis [41]. Reduced SIRT1 function contributes to mitochondrial dysfunction, increased oxidative stress, and activation of inflammatory pathways, including inflammasome signaling [29,30,32,34]. In addition, endoplasmic reticulum (ER) stress plays a critical role in DCM progression. SIRT1 has been shown to alleviate ER stress and suppress inflammatory signaling cascades, thereby protecting cardiomyocytes from metabolic injury [30,38]. Unlike I/R injury, where ferroptosis dominates, DCM is characterized by chronic metabolic imbalance and inflammatory activation, with SIRT1 acting as a key regulator of NAD^+^-dependent metabolic adaptation.

### 3.4. Cardiac Hypertrophy and Fibrosis

Cardiac hypertrophy and fibrosis are key pathological processes driven by pressure overload, hypertension, and neurohormonal activation, ultimately contributing to heart failure progression. In this setting, structural remodeling rather than acute stress responses represents the dominant pathological feature. SIRT1 modulates hypertrophic and fibrotic signaling by influencing transcriptional regulation, stress responses, and autophagy-related processes. Through these mechanisms, SIRT1 contributes to the suppression of maladaptive cardiac growth and extracellular matrix accumulation, consistent with the regulatory framework outlined in Section 2. In addition, SIRT1-mediated regulation of cellular quality control processes supports the maintenance of myocardial structure under sustained stress. These findings suggest that SIRT1 functions as an integrative regulator of cardiac remodeling, with potential implications for both myocardial and cardiorenal fibrosis.

### 3.5. Cardiac Aging

Cardiac aging is associated with progressive decline in mitochondrial function, increased oxidative stress, and impaired cellular repair mechanisms. A hallmark of aging is the gradual reduction in intracellular NAD^+^ levels, leading to diminished SIRT1 activity [5,7]. This decline contributes to impaired mitochondrial biogenesis, accumulation of oxidative damage, and reduced cellular resilience. Emerging evidence also highlights the role of SIRT1 in circadian rhythm regulation, which is closely linked to cardiac metabolism and aging [5,12,50]. Disruption of circadian control further exacerbates metabolic dysfunction and accelerates cardiac aging. In this context, SIRT1 functions as a central integrator of metabolic and temporal regulation, linking NAD^+^ availability, mitochondrial health, and circadian homeostasis.

### 3.6. SIRT1 and Circadian Rhythm (CR) Regulation

CR regulation is a fundamental determinant of cardiovascular physiology, influencing heart rate, blood pressure, metabolism, and vascular function. The circadian clock and SIRT1 form a reciprocal regulatory loop with direct implications for cardiovascular health [10]. SIRT1 deacetylates core clock components such as BMAL1 and PER2, modulating their oscillatory activity, while CLOCK/BMAL1 heterodimers regulate SIRT1 expression. Disruption of this loop, as occurs in shift work or aging, attenuates SIRT1 activity and exacerbates oxidative stress, endothelial dysfunction, and cardiac remodeling. Melatonin, a circadian regulator, has been shown to exert cardioprotection partly through restoring SIRT1 signaling [51]. The SIRT1–circadian axis therefore represents a promising, underexplored therapeutic target for cardiovascular diseases driven by chronodisruption.

## 4. Therapeutic Strategies Targeting SIRT1 in Cardiac Diseases

**Figure 3:**
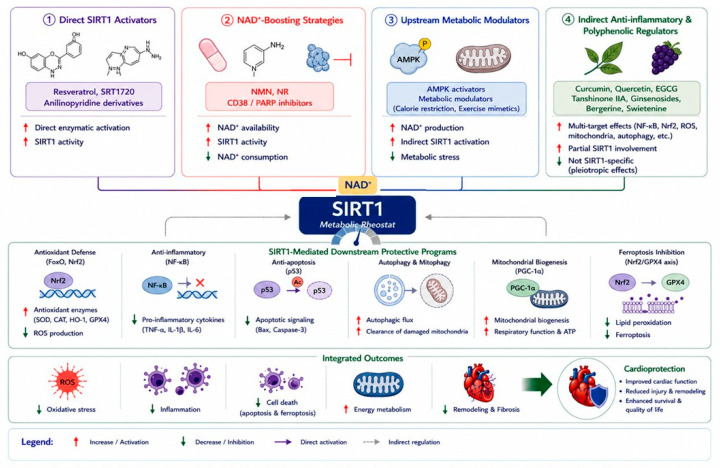
Mechanism-based therapeutic strategies targeting SIRT1 in cardiac diseases. Therapeutic strategies regulate SIRT1 via direct activation, NAD^+^ boosting, upstream metabolic modulation, and polyphenolic regulation. These approaches converge on shared downstream pathways, including antioxidant defense, anti-inflammatory effects, autophagy activation, and mitochondrial function. Together, they reduce oxidative stress and cell death while improving cardiac remodeling and function, ultimately contributing to cardioprotection.

To better reflect the complexity of SIRT1 signaling, therapeutic strategies are organized according to underlying mechanisms rather than individual compounds. This approach provides a more integrative understanding of how SIRT1-targeting interventions operate within interconnected metabolic and signaling networks (Figure 3).

### 4.1. Direct SIRT1 Activators

Direct activators of SIRT1 enhance its enzymatic activity through allosteric modulation or direct binding. Representative compounds such as resveratrol and synthetic activators (e.g., SRT1720) have demonstrated cardioprotective effects in various experimental models [14,25,26,52]. These agents improve mitochondrial function, reduce oxidative stress, and enhance cellular survival pathways. However, their efficacy may depend on cellular context and dosage, and variability in bioavailability remains a challenge. Therefore, while direct activation represents a promising strategy, its clinical translation requires further optimization.

### 4.2. NAD^+^-Boosting Strategies

Given that SIRT1 activity is dependent on NAD^+^ availability, increasing intracellular NAD^+^ levels represents an effective indirect approach to enhance SIRT1 function. NAD^+^ precursors, such as nicotinamide mononucleotide (NMN) and nicotinamide riboside (NR), have been shown to restore metabolic balance, improve mitochondrial function, and protect against cardiac injury [41,53,54]. In addition, modulation of NAD^+^-consuming enzymes, such as CD38 and poly(ADP-ribose) polymerases (PARPs), can further increase NAD^+^ bioavailability. This strategy targets upstream metabolic regulation and may provide more stable and sustained activation of SIRT1 compared to direct activators.

### 4.3. Upstream Metabolic Modulators

SIRT1 activity is also regulated by upstream signaling pathways, particularly those involved in energy sensing. Activation of AMP-activated protein kinase (AMPK) and related pathways can indirectly enhance SIRT1 activity by increasing NAD^+^ levels and improving metabolic efficiency [11,40]. This category includes interventions that modulate cellular energy status, thereby influencing SIRT1-dependent signaling as part of a broader metabolic network. Such approaches may offer advantages in terms of physiological relevance and systemic effects.

### 4.4. Indirect Anti-Inflammatory and Polyphenolic Regulators

A wide range of natural compounds, including polyphenols and plant-derived molecules, exert cardioprotective effects through multi-target mechanisms [25,30,38,55,56]. These agents modulate oxidative stress, inflammation, and metabolic pathways, with SIRT1 representing one of several downstream effectors. Importantly, the effects of these compounds cannot be attributed solely to SIRT1-mediated mechanisms, as they also influence pathways such as NF-κB and Nrf2. Their pleiotropic nature highlights the complexity of pharmacological modulation in cardiovascular diseases and underscores the need for careful interpretation of mechanistic studies.

## 5. SIRT1 as a Biomarker in Cardiac Diseases

**Figure 4:**
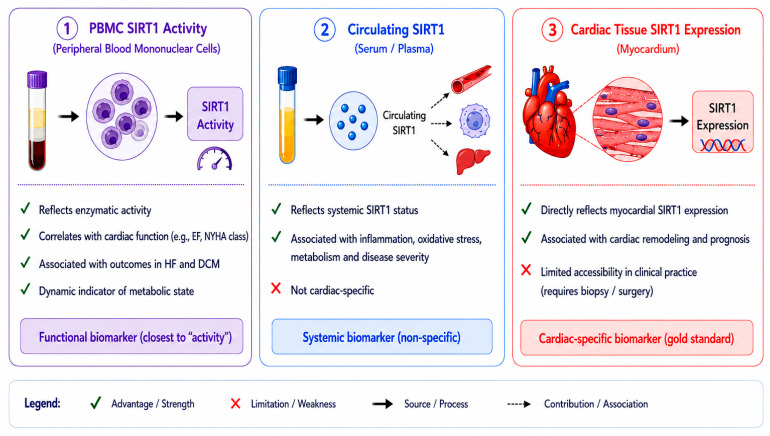
SIRT1 as a multi-dimensional biomarker in cardiac diseases. SIRT1 can be evaluated through PBMC activity, circulating levels, and myocardial expression. PBMC activity reflects enzymatic function, circulating SIRT1 indicates systemic status, and tissue expression provides cardiac-specific information. While circulating measures lack specificity and tissue sampling is invasive, these complementary readouts together enable comprehensive assessment of SIRT1 in cardiac diseases.

SIRT1 has emerged as a potential biomarker in cardiovascular diseases; however, its interpretation requires careful distinction among different biological readouts. SIRT1 activity measured in peripheral blood mononuclear cells (PBMCs) reflects systemic enzymatic function and has been associated with cardiac functional parameters such as ejection fraction [57]. Serum or plasma SIRT1 concentrations represent circulating protein abundance, which may reflect systemic metabolic or inflammatory status rather than direct cardiac expression [58]. Cardiac tissue expression of SIRT1 provides the most direct indication of local biological activity but is less accessible in clinical settings [59]. Importantly, these measures are not interchangeable and may reflect distinct physiological or pathological processes. Therefore, caution is required when interpreting SIRT1 as a biomarker across different clinical contexts (Figure 4).

## 6. Challenges and Unresolved Questions

Despite extensive preclinical evidence, several challenges hinder clinical translation of SIRT1-targeted therapies for cardiac diseases.

### 6.1. Dose-Dependent Dual Role

SIRT1 exhibits a biphasic effect: low-to-moderate activation is protective, whereas excessive activation can be detrimental. In pressure overload-induced HF, SIRT1 upregulation was associated with contractile dysfunction, and SIRT1 knockout was protective [15]. Similarly, high-level (12.5-fold) SIRT1 overexpression induced cardiomyopathy, while moderate (2.5- to 7.5-fold) overexpression was beneficial [13]. Therefore, achieving optimal SIRT1 activation levels—rather than maximal activation—is critical.

### 6.2. Cardiac Specificity

Systemic SIRT1 activation may affect other organs (liver, adipose tissue, brain), potentially causing unintended metabolic or neurological effects. SIRT1 regulates hepatic gluconeogenesis, adipocyte lipolysis, and appetite [7]. Thus, cardiac-specific delivery strategies (e.g., nanoparticle-based targeting, adeno-associated virus vectors, or RNA-based approaches) are needed. Recent advances in gene-activating tetrahedral DNA nanoparticles targeting SIRT1 have shown promise in fibrotic models and could be adapted for cardiac applications.

### 6.3. NAD^+^ Dependency and Aging

SIRT1 activity declines with age due to reduced NAD^+^ levels [2,7]. NAD^+^ supplementation may restore SIRT1 function, but whether this is sufficient to reverse established cardiac pathology is unclear. Moreover, NAD^+^ is consumed by other enzymes (PARPs, CD38), and competition for NAD^+^ may limit SIRT1 activation. Combinatorial strategies that inhibit CD38 or PARP1 while boosting NAD^+^ could be more effective.

### 6.4. Stage-Specific Effects

The mechanisms driving early cardiac dysfunction differ from those in end-stage HF. SIRT1 activation may be most beneficial during early stress adaptation (e.g., after MI or during metabolic dysfunction) but less effective once irreversible remodeling and fibrosis have occurred. Identifying optimal therapeutic windows is essential.

### 6.5. Translational Gap

Most studies are in rodent models, which do not fully recapitulate human cardiac disease heterogeneity. Large animal studies and well-designed clinical trials are lacking. Several natural compounds (resveratrol, quercetin) have undergone clinical trials for metabolic and cardiovascular endpoints, but results have been inconsistent, possibly due to poor bioavailability and lack of SIRT1 selectivity. Synthetic activators (SRT1720, SRT2104) have entered human trials for metabolic diseases, but cardiac outcomes have not been specifically evaluated.

### 6.6. Methodological and Mechanical Limitations

Despite the large body of evidence supporting a cardioprotective role for SIRT1, several important limitations must be acknowledged when interpreting the available data and when designing future studies.

#### 6.6.1. Pharmacological Off-Target Effects

A substantial proportion of studies, particularly those investigating natural compounds, rely on resveratrol as a SIRT1 activator. However, resveratrol is not a selective SIRT1 agonist; it also activates AMPK, inhibits cyclooxygenases, modulates estrogen receptors, and affects multiple other signaling pathways [14]. Consequently, cardioprotective effects observed with resveratrol cannot be unequivocally attributed to SIRT1 activation. Similarly, many other natural polyphenols and flavonoids (e.g., quercetin, curcumin) have broad biological activities that extend beyond SIRT1 [55,60]. Even synthetic activators such as SRT1720 and SRT2104 may have off-target effects at higher concentrations [56]. Future studies should therefore employ complementary approaches, including genetic manipulation (knockdown, knockout, or overexpression) alongside pharmacological activation, to establish causality.

#### 6.6.2. Genetic Model Limitations

Cardiomyocyte-specific or inducible SIRT1 knockout and transgenic overexpression models have provided invaluable mechanistic insights. However, constitutive genetic modifications may induce developmental or compensatory adaptations that confound interpretation. For example, lifelong SIRT1 deficiency could upregulate other sirtuins (SIRT2, SIRT3, SIRT6) or alternative stress-response pathways, potentially masking the true role of SIRT1 in adult disease [13,22]. Conversely, chronic SIRT1 overexpression may lead to secondary metabolic changes that are not relevant to acute or subacute cardioprotection. Inducible and cell-type-specific systems (e.g., Cre-ERT2 under a cardiomyocyte-specific promoter) offer improved temporal and spatial control and should be more widely adopted.

#### 6.6.3. Measurement of SIRT1 Activity Versus Expression

A recurring limitation in the literature is the use of SIRT1 protein or mRNA levels as a proxy for its enzymatic activity. SIRT1 is a NAD^+^-dependent deacetylase, and its activity is profoundly influenced by cellular NAD^+^ availability, which varies with age, metabolic state, and disease [7,61]. High SIRT1 expression does not guarantee high deacetylase activity if NAD^+^ levels are depleted. Conversely, low expression may be compensated by increased NAD^+^ or by post-translational modifications that enhance specific activity. Direct assessment of SIRT1 deacetylase activity—for example, using fluorometric activity assays, acetylated substrate detection (e.g., acetyl-p53, acetyl-FoxO1, or acetyl-PGC-1α), or activity-based probes—should be incorporated into future studies.

#### 6.6.4. Incomplete Recapitulation of Human Disease

The vast majority of mechanistic studies have been conducted in rodent models (mice and rats), which do not fully reproduce the complexity, heterogeneity, and chronicity of human cardiac diseases. Murine hearts have different metabolic rates, ion channel profiles, and contractile properties compared to human hearts. Moreover, most experimental models involve young, healthy, genetically homogeneous animals, whereas human patients with HF or DCM are typically elderly, have multiple comorbidities, and take numerous concomitant medications. The translational failure of many cardioprotective agents that were successful in animal models underscores this limitation [62,63]. Larger animal models (e.g., pigs, dogs) and human tissue studies (e.g., explanted hearts, endomyocardial biopsies) are needed to validate SIRT1-targeted strategies.

#### 6.6.5. Lack of Standardized Protocols and Outcome Measures

Across the literature, there is considerable heterogeneity in SIRT1 activator dosing regimens, routes of administration, treatment durations, and outcome assessments. For example, resveratrol doses range from 5 to 100 mg/kg/day in different rodent studies, with variable bioavailability and pharmacokinetics [14,25,26]. Similarly, studies use different models of I/R injury (in vivo ligation vs. ex vivo Langendorff), different ischemic times, and different reperfusion durations, making direct comparisons difficult. Standardization of experimental protocols and adoption of core outcome sets would improve reproducibility and facilitate meta-analyses.

#### 6.6.6. Publication Bias and Positive Outcome Bias

As in many fields of biomedical research, studies reporting positive or protective effects of SIRT1 activation are more likely to be published than those showing neutral or detrimental effects. The well-known dual role of SIRT1, where moderate activation is beneficial but excessive activation is harmful, may be under-reported [6,13,15]. Negative findings or dose–response studies that reveal a narrow therapeutic window are equally important for guiding safe translation. Journals and funding agencies should encourage the publication of well-conducted studies regardless of the direction of results.

#### 6.6.7. Limited Clinical Validation

Despite decades of preclinical research, few SIRT1-targeted interventions have progressed to randomized controlled trials with hard cardiovascular endpoints. Most human studies have been small, short-term, and focused on surrogate biomarkers (e.g., flow-mediated dilation, inflammatory markers) rather than clinical outcomes such as death, HF hospitalization, or major adverse cardiovascular events [57,58]. The few trials that have been conducted with resveratrol or NAD^+^ precursors have yielded mixed results, possibly due to poor bioavailability, inadequate dosing, or inappropriate patient selection. Rigorous, adequately powered phase 2 and phase 3 trials are urgently needed to determine whether modulation of SIRT1-dependent signaling can improve outcomes in patients with HF, post-infarction remodeling, or DCM.

#### 6.6.8. Overlooked Cell-Type and Context Specificity

Most studies examine SIRT1 effects in a single cell type (cardiomyocytes, endothelial cells, or macrophages) in isolation. However, the heart is a complex organ with extensive cross-talk among different cell populations. SIRT1 activation in cardiomyocytes may have different effects than SIRT1 activation in cardiac fibroblasts, endothelial cells, or resident immune cells. Moreover, the role of SIRT1 may differ between the acute phase of injury (e.g., I/R) and the chronic phase of remodeling and failure. Single-cell transcriptomic and spatial omics approaches are needed to dissect these cell-type- and stage-specific functions [64].

#### 6.6.9. Interaction with Other Sirtuins

SIRT1 is only one member of the sirtuin family. SIRT3 (mitochondrial), SIRT6 (nuclear), and others also play important roles in cardiac protection and may compensate for or interact with SIRT1 [65,66]. For instance, Exendin-4 protects against I/R injury by upregulating both SIRT1 and SIRT3 [65]. Isolating the specific contribution of SIRT1 from other sirtuins is challenging, particularly with NAD^+^-boosting strategies that affect all NAD^+^-dependent enzymes. Future studies should employ isoform-selective activators or genetic models that allow dissection of individual sirtuin contributions. In addition to SIRT3 and SIRT6, other members of the sirtuin family, particularly SIRT7, have also emerged as important regulators of cardiovascular homeostasis [67,68]. SIRT7 has been implicated in maintaining cardiac structure and function, and its deficiency has been associated with increased susceptibility to cardiac stress and dysfunction. Importantly, accumulating evidence suggests the existence of functional cross-talk between SIRT1 and SIRT7. SIRT7 can modulate SIRT1 activity by influencing its acetylation status and downstream signaling, thereby contributing to the fine-tuning of cellular differentiation, stress responses, and metabolic regulation [68]. This interaction highlights the coordinated regulation within the sirtuin family and suggests that SIRT1-mediated effects in cardiac diseases may be partly shaped by its interplay with SIRT7. These findings emphasize that sirtuin family members act in an integrated network rather than in isolation, and that understanding their interactions may provide new insights into the mechanisms of cardioprotection and therapeutic targeting.

#### 6.6.10. Temporal Resolution Problem

A notable limitation in current SIRT1 research is the lack of temporal resolution in assessing its activity. Most studies rely on endpoint measurements, such as protein expression levels or downstream signaling readouts, which may fail to capture the dynamic and transient nature of SIRT1 activation. As recently highlighted, the actual response to SIRT1/AMPK signaling in the heart is critically determined by the intensity and duration of its activation—transient, measured signaling produces adaptive effects, whereas continuous heightened activity yields maladaptive responses [69]. Yet, very few studies have tracked SIRT1 activity longitudinally across disease progression. In acute conditions such as I/R, SIRT1 expression and activity undergo rapid changes within hours of reperfusion [22,70]. Similarly, in chronic conditions, prolonged activation or suppression may lead to distinct biological outcomes, including the dose- and duration-dependent effects observed in cardiac remodeling. Therefore, future studies should incorporate real-time or longitudinal monitoring approaches, such as genetically encoded NAD^+^/SIRT1 biosensors, time-resolved enzymatic activity assays, and dynamic NAD^+^ flux measurements, to better characterize the temporal dynamics of SIRT1 activity. Addressing this limitation will be essential for understanding the context-dependent roles of SIRT1 and for optimizing therapeutic strategies targeting this pathway.

#### 6.6.11. Cell-Type Specificity

Another important limitation is the insufficient consideration of cell-type-specific roles of SIRT1 within the cardiac microenvironment. While many studies focus on cardiomyocytes, SIRT1 also plays distinct roles in cardiac fibroblasts, endothelial cells, and immune cells. In cardiomyocytes, SIRT1 primarily regulates mitochondrial function, oxidative stress, and autophagic flux, thereby governing cell survival under stress [71]. In fibroblasts, SIRT1 suppresses myofibroblast differentiation and extracellular matrix deposition by deacetylating Smad2/3 and attenuating TGF-β signaling [72]. In endothelial cells, SIRT1 maintains vascular homeostasis and angiogenesis; for example, the Sirt1/Drp1 pathway protects cardiac microvascular endothelial cells from diabetic injury by inhibiting excessive mitochondrial division [73]. In immune cells, SIRT1 dynamically regulates inflammatory responses and macrophage M1/M2 polarization through metabolic reprogramming and epigenetic mechanisms, with SIRT1 deficiency promoting a pro-inflammatory M1 phenotype [74]. These diverse functions highlight that SIRT1-mediated signaling is highly cell-type dependent, and a more integrated, multicellular perspective is required to fully understand its role in cardiac diseases.

#### 6.6.12. NAD^+^ Competition

Given that SIRT1 activity is dependent on NAD^+^ availability, competition among NAD^+^-consuming enzymes represents a critical regulatory layer. Under pathological stress conditions, enzymes such as PARPs and CD38 become activated and consume substantial amounts of NAD^+^, thereby limiting substrate availability for SIRT1 [75]. This energetic competition can impair SIRT1 activity even in the presence of upstream activating signals. Consequently, the balance between NAD^+^ production and consumption plays a pivotal role in determining SIRT1 functionality. Targeting NAD^+^ metabolism, including inhibition of excessive PARP or CD38 activity, may therefore represent an effective strategy to restore SIRT1-dependent signaling in cardiovascular diseases.

In summary, while the existing evidence strongly supports SIRT1 as a critical regulator of cardiac health and disease, careful attention to these limitations is necessary to avoid overinterpretation and to guide the design of more rigorous, translatable studies. Addressing these methodological and conceptual gaps will be essential for moving SIRT1-targeted therapies from bench to bedside.

## 7. Future Directions

Despite the substantial progress made in understanding the cardioprotective roles of SIRT1, several critical challenges must be addressed before these findings can be translated into clinical practice. One of the most pressing issues is the context-dependent dual role of SIRT1 in the heart. Preclinical studies have clearly demonstrated that low-to-moderate SIRT1-dependent signaling engagement confers protection against I/R injury, HF, and DCM, whereas excessive SIRT1 overexpression or chronic hyperactivation can exacerbate cardiac dysfunction and even induce cardiomyopathy [6,13,15]. Therefore, future therapeutic strategies should aim for precise, dose-controlled modulation of SIRT1 rather than maximal or indiscriminate SIRT1 pathway activation. This will require the development of allosteric activators that fine-tune SIRT1 deacetylase activity without increasing its protein expression, as well as inducible or short-acting pharmacological agents that allow temporal control of SIRT1 signaling.

Another major hurdle is the lack of cardiac specificity in current SIRT1-targeted approaches. Because SIRT1 is ubiquitously expressed and regulates fundamental metabolic processes in the liver, adipose tissue, and brain, systemic activation may lead to unintended metabolic or neurological side effects [7]. To overcome this limitation, future research should prioritize cell-type-specific delivery strategies. For example, nanoparticle-encapsulated SIRT1 mRNA, adeno-associated virus vectors with cardiomyocyte-specific promoters, or endothelium-targeted liposomes could achieve localized SIRT1 modulation within the heart. Recent advances in gene-activating technologies, such as tetrahedral DNA nanoparticles that restore SIRT1 expression in a tissue-restricted manner, offer promising templates that could be adapted for cardiac applications.

The dependence of SIRT1 on NAD^+^ as an essential cofactor introduces another layer of complexity. Aging, obesity, and chronic inflammation all deplete cellular NAD^+^ levels, thereby limiting SIRT1 activity even when the protein is adequately expressed [2,7]. While NAD^+^ precursors such as nicotinamide riboside and nicotinamide mononucleotide have shown efficacy in preclinical models, it remains uncertain whether simple NAD^+^ repletion is sufficient to restore SIRT1 function in patients with established cardiac disease [41,61]. Moreover, NAD^+^ is also consumed by other enzymes, including PARPs and CD38, which compete with SIRT1 for the same pool. Future studies should therefore explore combinatorial strategies that simultaneously boost NAD^+^ availability and inhibit NAD^+^-consuming enzymes, thereby channeling NAD^+^ toward SIRT1-dependent protective pathways.

It is also becoming clear that the beneficial effects of SIRT1 activation are likely stage-dependent. Early after myocardial infarction or during the initial phases of metabolic stress, enhancing SIRT1 activity may help limit oxidative damage, preserve mitochondrial function, and prevent maladaptive remodeling. However, once irreversible fibrosis, extensive cardiomyocyte loss, or end-stage HF has developed, SIRT1 activation may no longer confer meaningful benefits. Identifying optimal therapeutic windows will require longitudinal studies in animal models that recapitulate the different phases of human cardiac disease, as well as the development of non-invasive biomarkers that can monitor SIRT1 activity in real time.

The translation of SIRT1-targeted therapies into clinical practice is further hampered by the limitations of current preclinical models. Most studies have been conducted in young, healthy rodents, which do not fully reflect the age-related NAD^+^ decline, multimorbidity, and medication burden seen in elderly cardiac patients. Large-animal studies and well-designed clinical trials are urgently needed. Several natural SIRT1 activators, including resveratrol and quercetin, have already entered human trials for metabolic and cardiovascular endpoints, but results have been inconsistent, largely due to poor bioavailability and lack of target selectivity [55]. Synthetic activators such as SRT1720 and SRT2104 have shown promise in early-phase studies, but their effects on hard cardiac outcomes (e.g., mortality, HF hospitalization) have not been rigorously evaluated. Future clinical trials should enroll well-defined patient populations (e.g., post-MI with reduced ejection fraction, DCM, or HF with preserved ejection fraction), incorporate direct or surrogate measures of SIRT1 activity in peripheral blood mononuclear cells or cardiac tissue, and use a combination of functional, biomarker, and clinical endpoints.

Methodological improvements are also essential. Many studies still rely on resveratrol as a SIRT1 activator, yet resveratrol has multiple off-target effects, including AMPK activation and estrogen receptor modulation, which confound interpretation [14]. Genetic models, while more specific, may induce developmental compensation that does not reflect adult disease dynamics. Furthermore, SIRT1 expression levels are often used as a surrogate for its activity, but enzymatic activity depends critically on NAD^+^ availability and post-translational modifications. Direct measurement of SIRT1 deacetylase activity in cardiac tissue, for instance, using activity-based probes or acetylated substrate assays, should become standard in future mechanistic studies.

Looking ahead, the integration of multi-omics technologies and artificial intelligence offers unprecedented opportunities to dissect SIRT1-dependent regulatory networks in a cell-type-specific and disease-stage-specific manner [64,76]. Single-cell transcriptomics can reveal how SIRT1 modulates distinct cardiomyocyte, fibroblast, endothelial, and immune cell populations within the diseased heart. Proteomic and metabolomic analyses can identify novel SIRT1 substrates and downstream metabolic signatures. Machine learning models trained on large clinical datasets may help identify patient subgroups that are most likely to benefit from SIRT1-targeted interventions, thereby enabling a precision medicine approach.

Finally, non-coding RNAs represent an emerging frontier for SIRT1 modulation. Several microRNAs (e.g., miR-448, miR-489-3p, miR-199a-5p) and long non-coding RNAs (e.g., HAND2-AS1, lincRNA-p21) have been shown to directly regulate SIRT1 expression in cardiac cells [77,78]. Antagomirs targeting these inhibitory microRNAs, or synthetic mimics of SIRT1-enhancing long non-coding RNAs, could provide a new class of SIRT1-modulating therapeutics. For example, ginsenoside Rg1 was found to promote wound healing in diabetic models by downregulating miR-489-3p and upregulating SIRT1, suggesting that combination therapies involving natural compounds and RNA-based agents may achieve synergistic effects [79].

In summary, the future of SIRT1-based cardioprotection lies not in simple, one-size-fits-all activation, but in a nuanced, precision-oriented approach that considers dose, duration, cell type, disease stage, metabolic context, and individual patient variability. By embracing these complexities and leveraging modern technologies in drug delivery, multi-omics, and artificial intelligence, SIRT1 can transition from a compelling mechanistic target to a clinically transformative node in precision cardiovascular therapy.

## 8. Conclusions

**Figure 5:**
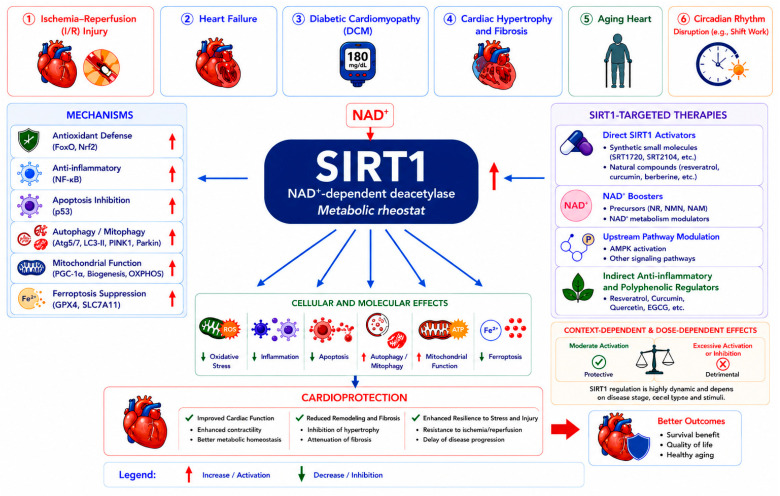
SIRT1-mediated cardioprotection across disease contexts, mechanisms and therapies. SIRT1 functions as a central metabolic regulator in diverse cardiac diseases, including ischemia–reperfusion injury, heart failure, diabetic cardiomyopathy, cardiac hypertrophy and fibrosis, aging-related cardiac dysfunction, and circadian rhythm disruption. Activated by NAD^+^, SIRT1 modulates multiple protective mechanisms, such as antioxidant defense, anti-inflammatory signaling, inhibition of apoptosis, promotion of autophagy and mitophagy, enhancement of mitochondrial function, and suppression of ferroptosis. Therapeutic strategies targeting SIRT1 include direct activators, NAD^+^-boosting approaches, upstream pathway modulation, and indirect anti-inflammatory and polyphenolic regulators. These interventions converge on shared cellular and molecular effects, leading to reduced oxidative stress, inflammation, and cell death, alongside improved mitochondrial function and metabolic homeostasis. Notably, SIRT1 activity exhibits context- and dose-dependent effects, where moderate activation is protective, whereas excessive activation or inhibition may be detrimental. Collectively, SIRT1-mediated pathways contribute to cardioprotection by improving cardiac function, limiting adverse remodeling and fibrosis, enhancing stress resilience, and ultimately promoting better clinical outcomes and healthy aging.

SIRT1 functions as a central metabolic–inflammatory integrator in the heart, coordinating antioxidant defense, anti-inflammatory signaling, apoptosis suppression, autophagic flux, mitochondrial biogenesis, and ferroptosis control. Across a spectrum of cardiac diseases, including I/R injury, HF, DCM, cardiac hypertrophy, and aging, SIRT1-dependent regulatory networks exert protective effects, albeit with a narrow therapeutic window where excessive activation becomes harmful (Figure 5).

Preclinical evidence from natural compounds, synthetic activators, NAD^+^ precursors, and lifestyle interventions consistently demonstrates that SIRT1 is a druggable target for cardioprotection. However, clinical translation remains hindered by challenges in achieving cardiac specificity, optimal dosing, and validation in human populations.

Future research should move beyond reductionist activation strategies and embrace precision medicine approaches that consider disease stage, metabolic context, cell-type specificity, and individual genetic variability. Integrating multi-omics technologies, AI-driven risk stratification, and advanced drug delivery systems will be essential to unlock the full therapeutic potential of SIRT1 in cardiac diseases. As our understanding of SIRT1 biology deepens, this NAD^+^-dependent deacetylase may transition from a compelling mechanistic target to a clinically transformative node in precision cardiovascular therapy.

## Data Availability

No new data were created or analyzed in this study. Data sharing is not applicable to this article.

## Abbreviations

SIRT1: Sirtuin 1; NAD^+^: Nicotinamide adenine dinucleotide; CVDs: Cardiovascular diseases; I/R: Ischemia/reperfusion; HF: Heart failure; DCM: Diabetic cardiomyopathy; ROS: Reactive oxygen species; NF-κB: Nuclear factor kappa B; Nrf2: Nuclear factor erythroid 2-related factor 2; FoxO: Forkhead box O; PGC-1α: Peroxisome proliferator-activated receptor gamma coactivator 1-alpha; GPX4: Glutathione peroxidase 4; HO-1: Heme oxygenase-1; NQO1: NAD(P)H quinone oxidoreductase 1; AMPK: AMP-activated protein kinase; NR: Nicotinamide riboside; NMN: Nicotinamide mononucleotide; PBMCs: Peripheral blood mononuclear cells; MACE: Major adverse cardiac events; MALE: Major adverse limb events; ER: Endoplasmic reticulum; PARPs: Poly(ADP-ribose) polymerases.
